# Daphmacrodins A and B, alkaloids from *Daphniphyllum macropodum*

**DOI:** 10.1007/s13659-012-0095-z

**Published:** 2013-02-26

**Authors:** Ming-Ming Cao, Hong-Ping He, Yu-Cheng Gu, Qiang Zhang, Xiao-Nian Li, Guo-Ying Zuo, Ying-Tong Di, Chun-Mao Yuan, Shun-Lin Li, Yu Zhang, Xiao-Jiang Hao

**Affiliations:** 195 State Key Laboratory of Phytochemistry and Plant Resources in West China, Kunming Institute of Botany, Chinese Academy of Sciences, Kunming, 650201 China; 295 University of Chinese Academy of Sciences, Beijing, 100049 China; 395 Syngenta, Jealott’s Hill International Research Centre, Bracknell, Berkshire, RG42 6EY UK; 495 Northwest A&F University, Yangling, 712100 China; 595 Research Center of Natural Medicine, Clinical School of Kunming General Hospital of Chengdu Military Command, Kunming, 650032 China

**Keywords:** Daphniphyllaceae, *Daphniphyllum macropodum*, cyclopentadienyl anion

## Abstract

Two new daphnicyclidin-type *Daphniphyllum* alkaloids, daphmacrodins A and B (**1** and **2**) were isolated from the leaves and stems of *Daphniphyllum macropodum*. Their structures were elucidated by extensive spectroscopic techniques, including 2D NMR spectroscopy and mass spectrometry. The relative configuration of **1** was further confirmed by a single-crystal X-ray diffraction analysis. Their cytotoxic activities against five human cancer cell lines, pesticidal activities against brine shrimp (*Artemia salina*), and antibacterial activities against five standard bacterial and fungal strains were evaluated. The structure of **1** was successfully transformed to **2** by a chemical method.

**Electronic Supplementary Material:**

Supplementary material is available for this article at 10.1007/s13659-012-0095-z and is accessible for authorized users.

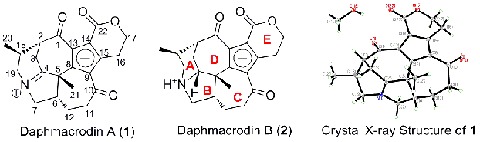

## Introduction

*Daphniphyllum* alkaloids are a family of diversified and complex polycyclic natural products elaborated by plants of the genus *Daphniphyllum*.[1] Their unique structural features have attracted great interest as challenging targets for total synthesis[2] and biosynthetic research[3] for several decades. In recent years, quite a number of new *Daphniphyllum* alkaloids have been isolated and identified, and some of them possessed novel skeletons.[4] In our continuing search for structurally unique *Daphniphyllum* alkaloids,[4b–4d] two new daphnicyclidin-type compounds,[5] daphmacrodins A and B (**1** and **2**) with rare cyclopentadienyl anion[6] were isolated from the leaves and stems of *D. macropodum*. We report herein the isolation, structural elucidation, and the bioactivities of these two alkaloids.

## Results and Discussion

Daphmacrodin A (**1**) was obtained as colorless monoclinic crystals (MeOH) with $[\alpha]^{22}_{D}$ + 155 (*c* 0.19, MeOH). The molecular formula, $C_{22}H_{23}NO_4$, was established by positive HRESIMS (*m/z* 388.1528 $[M + Na]^+$, calcd for $C_{22}H_{23}NO_4Na$, 388.1524), corresponding to 12 degrees of unsaturation. The IR absorption spectrum showed the presence of conjugated carbonyl (1668 and 1600 $cm^{-1}$) functionality. The $^{13}C$ NMR and DEPT data (Table 1) revealed 22 carbon signals comprising six $sp^2$ carbon atoms (including one imine carbon atom), three carbonyl groups, and 13 $sp^3$ carbon atoms (1 × C, 3 × CH, 7 × $CH_2$, 2 × $CH_3$). Among them, two methylenes ($\delta_C$ 63.1 and 51.9) were ascribed as attached to a nitrogen atom, while one methylene ($\delta_C$ 66.2) was ascribed as bearing an oxygen atom. Since nine $sp^2$ quaternary carbons accounted for six out of 12 unsaturation, the remaining six degrees of unsaturation were assumed for the presence of a hexacyclic system in **1**.

**Figure 1.** Structures of **1** and **2**

*To whom correspondence should be addressed. E-mail: haoxj@mail.kib.ac.cn (X.J. Hao); zhangyu@mail.kib.ac.cn (Y. Zhang)

Springer

**Table 1.** $^{13}$C NMR spectroscopic data for daphmacrodins A and B (1 and 2)

| pos. | **1**[a] | **2**[a] | pos. | **1**[a] | **2**[a] |
|---|---|---|---|---|---|
| 1 | 193.6 C | 194.6 C | 12 | 25.8[b] $CH_2$ | 27.5 $CH_2$ |
| 2 | 52.0 CH | 47.1 CH | 13 | 121.9 C | 122.5 C |
| 3 | 25.8[b] $CH_2$ | 17.0 $CH_2$ | 14 | 112.1 C | 112.3 C |
| 4 | 196.7 C | 66.4 CH | 15 | 138.0 C | 139.1 C |
| 5 | 58.3 C | 49.9 C | 16 | 25.2 $CH_2$ | 25.3 $CH_2$ |
| 6 | 44.0 CH | 47.6 CH | 17 | 66.2 $CH_2$ | 65.8 $CH_2$ |
| 7 | 63.1 $CH_2$ | 59.3 $CH_2$ | 18 | 34.2 CH | 28.5 CH |
| 8 | 128.8 C | 133.5 C | 19 | 51.9 $CH_2$ | 52.9 $CH_2$ |
| 9 | 117.7 C | 120.5 C | 20 | 18.7 $CH_3$ | 16.7 $CH_3$ |
| 10 | 197.6 C | 198.8 C | 21 | 28.3 $CH_3$ | 35.1 $CH_2$ |
| 11 | 39.5 $CH_2$ | 39.5 $CH_2$ | 22 | 161.8 C | 162.0 C |

[a]Recorded in DMSO-$d_6$ at 125 MHz
[b]Overlapped

Detailed analysis of 2D NMR (HSQC, $^1$H-$^1$H COSY, and HMBC experiments, Fig. 2) revealed that **1** had a daphnicyclidin-type skeleton and possessed three spin coupling systems, ***a***: H-3/H-2/H-18/H-19 (Me-20), ***b***: H-7/H-6/H-12/H-11 and ***c***: H-16/H-17 (Fig. 2A). In the HMBC spectrum, cross-peaks of H-19 ($\delta_H$ 4.00, dd, $J$ = 14.0, 7.5 Hz; $\delta_H$ 3.20, dd, $J$ = 14.0, 9.0 Hz) to C-7 ($\delta_C$ 63.1) indicated that the fragments ***a*** and ***b*** were connected through the nitrogen atom. The linkages of C-21, C-4, C-6, and C-8 to C-5 were fixed by the HMBC correlations from Me-21 ($\delta_H$ 1.69, s) to C-5 ($\delta_C$ 58.3), C-6 ($\delta_C$ 44.0) and C-8 ($\delta_C$ 128.8); H-6 ($\delta_H$ 2.80, m) to C-5 and C-8; H-3 ($\delta_H$ 3.57, dd, $J$ = 13.5, 2.5 Hz; $\delta_H$ 2.74, dd, $J$ = 13.5, 2.5 Hz) to C-4 ($\delta_C$ 196.7) and C-5 . Moreover, the HMBC correlations from H-11 ($\delta_H$ 2.37, dd, $J$ = 18.5, 6.5 Hz) to C-9 (117.7) and H-16 (2.65, m) to C-9 revealed that fragments ***b*** and ***c*** were linked through C-10-C-9-C-15 fragment. The attachment of C-1 to C-2 as well as C-11 to C-10 were established by HMBC cross-peaks of H-2 ($\delta_H$ 2.86, m) to C-1 ($\delta_C$ 193.6) and H-11 ($\delta_H$ 2.63, m) to C-10 ($\delta_C$ 197.6), respectively. Ring E was fused to rings C and D through the cyclopentadienyl anion ring was eventually resolved by the X-ray diffraction analysis (Fig. 3). Thus, the planar structure of **1** was established as a hexacyclic framework (Fig. 1). The $\beta$ orientation of H-2, Me-20, Me-21 and H-6 was deduced from ROESY correlations of Me-20/H-2, Me-20/H-3b, H-3b/Me-21, Me-21/H-6, which was further confirmed by the results of single-crystal X-ray diffraction analysis.

**Figure 2.** $^1$H-$^1$H COSY (Bold) and key HMBC correlations of **1** (A) and **2** (B)

Daphnicyclidin B (**2**), possesses a molecular formula of $C_{22}H_{25}NO_4$ determined by positive HRESIMS ($m/z$ 368.1861 [M + H]$^+$, calcd for $C_{22}H_{26}NO_4$, 368.1861) and 11 degrees of unsaturation. The NMR data showed high similarity to those of **1**, except that the quaternary carbon C-4 ($\delta_C$ 196.7) was replaced by a methine carbon at $\delta_C$ 66.4. This assignment was confirmed by $^1$H-$^1$H COSY and HMBC spectra (Fig. 2), especially by the HMBC correlations from H-7 ($\delta_H$ 3.81, t, $J$ = 11.0 Hz; 2.73, m) and H-19 ($\delta_H$ 3.16, m) to C-4 ($\delta_C$ 66.4) (Fig. 2B). The $\beta$-oriented H-4 ($\delta_H$ 3.53, br. s) was fixed by the ROESY correlations between H-4 and Me-21 ($\delta_H$ 1.36, s). Further detailed analysis of the 2D NMR (HSQC, $^1$H-$^1$H COSY, and HMBC experiments) revealed that the rest part of **2** was in agreement with **1**. Hence, the structure of **2** was established as shown in Fig. 1.

**Figure 3.** Crystal X-ray structure of **1**

To investigate the structural relationship between **1** and **2**, compound **1** was quantificationally transformed into **2** by reduction of the imine group in **1** according to reported method[7] (Scheme 1).

Compounds **1** and **2** were assayed *in vitro* for pesticidal activity against brine shrimp (*Artemia salina*) by the previously described Microwell method,[8] for their cytotoxicity against five human cancer cell lines (HL-60, SMMC-7721, A-549, MCF-7 and SW-480) using the MTT method[9] with cisplatin and paclitaxel as positive controls, and for bacteriostatic activity against (ATCC25923, ATCC27853, MRSA 92, and MRSA 98 by two-fold dilution method,[10] while no positive results were observed at concentrations of 50 and 100 $\mu$g/mL.

Daphmacrodin A (**1**) Daphmacrodin B (**2**)

**Scheme 1.** Reduction of **1** into **2**

## Experimental Section

**General Experimental Procedures.** Melting points were measured on a Yuhua X-4 digital microdisplaying Melting Point Apparatus. Optical rotations were measured with a Jasco P-1020 polarimeter. UV spectra were obtained using a Shimadzu UV-2401A spectrophotometer. CD spectra were recorded with an Applied Photophysics Chirascan spectrometer. A Tenor 27 spectrophotometer was used for IR spectra as KBr pellets. 1D and 2D NMR spectra were recorded on Bruker DRX-500 spectrometer with TMS as internal standard. HRESIMS was performed on an API QSTAR time-of-flight

**Table 2.** $^1$H NMR spectroscopic data for daphmacrodins A and B (1 and 2)

| pos. | 1[a] | 2[a] | pos. | 1[a] | 2[a] |
|---|---|---|---|---|---|
| 2 | 2.86 (m) | 2.48 (m) | 16 | 2.65 (m) | 3.01 (m); 2.54 (m) |
| 3 | 3.57 (dd, 13.5, 2.5); 2.74 (dd, 13.5, 5.5) | 2.21 (m) | 17 | 4.18 (m); 3.96 (m) | 4.11 (m) |
| 4 | | 3.53 (br. s) | 18 | 2.42 (m) | 2.23 (m) |
| 6 | 2.80 (m) | 2.48 (m) | 19 | 4.00 (dd, 14.0, 7.5); 3.20 (dd, 14.0, 9.0) | 3.16 (m) |
| 7 | 4.35 (dd, 13.5, 8.5); 3.63 (t, 11.0) | 3.81 (t, 11.0); 2.73 (m) | 20 | 1.20 (d, 7.0) | 1.17 (d, 7.5) |
| 11 | 2.63 (m); 2.37 (dd, 18.5, 6.5) | 2.50 (m); 2.37 (m) | 21 | 1.69 (s) | 1.36 (s) |
| 12 | 2.02 (m); 1.39 (m) | 1.99 (m); 1.64 (q, 13.0) | | | |

[a]Recorded in DMSO-$d_6$ at 500 MHz spectrometer. X-ray data were collected using a Bruker APEX DUO instrument. Semipreparative HPLC was performed on an Agilent 1100 liquid chromatograph with a Waters X-Bridge C18 (4.6 × 250 mm) column. Column chromatography (CC) were performed using silica (200–300 mesh and 300–400 mesh, Qingdao Marine Chemical, Inc., Qingdao, China) and Sephadex LH-20 (40–70 $\mu$m, Amersham Pharmacia Biotech AB, Uppsala, Sweden).

**Plant Material.** The leaves and stems of *D. macropodum* were collected from Sichuan Province, China, in October 2010. The plant samples were identified by Prof. Liangke Song of the School of Life Science and Engineering, Southwest Jiaotong University (SWJTU). A voucher specimen (KIB H20101011) was deposited at the State Key Laboratory of Phytochemistry and Plant Resource in West China, Kunming Institute of Botany, Chinese Academy of Science (CAS).

**Extraction and Isolation.** The air-dried, powdered leaves and stems (34 kg) of *D. macropodum* were extracted three times with 95% EtOH. The extract was adjusted with saturated tartaric acid to pH 2–3 and then defatted with petroleum ether (PE). Next, the aqueous phase was adjusted to pH 10 with saturated $Na_2CO_3$ and extracted with $CHCl_3$ to obtain the crude alkaloid fraction (260 g). The total alkaloid was subjected to normal phase Si gel (200–300 mesh; $CHCl_3$/MeOH, 1:0→0:1) to obtain five major fractions (Fr. 1–5). Fraction 4 (Fr. 4, 30 g) was further chromatographed over a reverse phase medium pressure column (MeOH/$H_2O$, 1:1→1:0) to give four fractions (Fr. 4A–4E). Fraction 4E (8.0 g) was subjected to normal phase Si gel (300–400 mesh; $CHCl_3$/MeOH = 25:1) to obtain seven fractions (Fr. 4E1–4E7). Fr. 4E7 was again separated by normal phase Si gel (300–400 mesh; $CHCl_3$/MeOH = 5:1) to give compounds **1** (30 mg) and **2** (4 mg).

**Daphmacrodin A (1):** colorless crystals; mp 136.0–137.0 °C; $[\alpha]_D^{22}$ + 155 (*c* 0.19, MeOH); UV (MeOH) $\lambda_{max}$ (log $\varepsilon$) 203 (3.33), 292 (3.55), 361 (3.17); CD (0.00026 M, MeOH) $\lambda_{max}$ ($\Delta\varepsilon$) 292 (–18.43) nm; IR (KBr) $\nu_{max}$ 3439, 2928, 1668, 1600, 1458, 1351, 1310, 1109, 1019 and 785 cm$^{-1}$; $^1$H and $^{13}$C NMR data, Tables 2 and 1; positive ESIMS *m/z* 366 $[M + H]^+$, 388 $[M + Na]^+$; positive HRESIMS $[M + Na]^+$ *m/z* 388.1528, calcd 388.1524.

**Daphmacrodin B (2):** white powder; $[\alpha]_D^{22}$ + 200 (*c* 0.07, MeOH); UV (MeOH) $\lambda_{max}$ (log $\varepsilon$) 203 (3.57), 297 (3.85), 362 (3.53); CD (0.00027 M, MeOH) $\lambda_{max}$ ($\Delta\varepsilon$) 292 (–16.69) nm; IR (KBr) $\nu_{max}$ 3428, 2927, 1659, 1607, 1455, 1348, 1232, 1076, 1047, 1018 and 785 cm$^{-1}$; $^1$H and $^{13}$C NMR data, Tables 2 and 1; positive ESIMS *m/z* 368 $[M + H]^+$, 390 $[M + Na]^+$; positive HRESIMS $[M + H]^+$ *m/z* 368.1861, calcd 368.1861.

**Reduction of 1 into 2:** A solution of **1** (5.0 mg) in anhydrous isopropanol (2 mL) was treated with sodium borohydride (1.6 mg, 3.08 eq.) The reaction mixture was stirred at 85 °C for 24 h in sealed tube. Then 0.1 mL saturated ammonium chloride aqueous was added and stirred for 1 h. The isopropanol was removed under diminished pressure and the residue was partitioned in $CHCl_3/H_2O$ = 1:1 (total 10 mL). The organic extracts were combined and the solvent was removed to give solid **2** (5.0 mg, 99.4%) which was determined by TLC analysis and negative ESIMS spectrum comparison with that of natural source of daphmacrodin B.

**X-ray Crystal Structure Analysis.** Colorless crystals of **1** were obtained from MeOH. Intensity data were collected at room temperature on a Bruker APEX DUO diffractometer equipped with an APEX II CCD using Mo K$\alpha$ radiation. Cell refinement and data reduction were performed with Bruker SAINT Software. The structure was solved by direct methods using SHELXL-97.[11] Refinements were performed with SHELXL-97 using full-matrix least-squares, with anisotropic displacement parameters for all the non-hydrogen atoms. The H-atoms were placed in calculated positions and refined using a riding model. Molecular graphics were computed with PLATON. Crystal data: $C_{23}H_{27}NO_5$ (containing one MeOH molecular), tetragonal space group $P2_1$, $a$ = 7.0149(9) Å, $b$ = 11.4350(14) Å, $c$ = 12.2778(15) Å, $\alpha$ = $\gamma$ = 90° $\beta$ = 95.218(2)°, $V$ = 980.8(2) Å$^3$, $Z$ = 2, $D_x$ = 1.346 kg m$^3$. Crystallographic data (excluding structure factor tables) for **1** were deposited with the Cambridge Crystallographic Data Center as supplementary publication No. CCDC 865586. Copies of the data can be obtained free of charge by application to CCDC, 12 Union Road, Cambridge CB 1EZ, UK [fax: Int. +44 (0) (1223) 336 033; e-mail: deposit@ccdc.cam.ac.uk]

## Electronic Supplementary Material

Supplementary material is available in the online version of this article at http://dx.doi.org/ 10.1007/s13659-012-0095-z and is accessible for authorized users.

## Acknowledgments

We thank Prof. Yan Li, State Key Laboratory of Phytochemistry and Plant Resources in West China, Kunming Institute of Botany, Chinese Academy of Sciences (KIB, CAS) for cytotoxicity testing. This work was financially supported by the National Natural Science Foundation of China

(30830114), the National Basic Research Program of China (973 Program, 2009CB522303). The Syngenta postgraduate studentship awarded to Mingming Cao (2012–2015) is appreciated.

**Open Access** This article is distributed under the terms of the Creative Commons Attribution License which permits any use, distribution, and reproduction in any medium, provided the original author(s) and source are credited.

## Electronic supplementary material

Supplementary material, approximately 4.91 MB.
