# Supplementary material for: Daphmacrodins A and B, alkaloids from Daphniphyllum macropodum
Source: Nat Prod Bioprospect. 2013 Feb 26;3(1):29–32. doi: 10.1007/s13659-012-0095-z (PMC4131615; doi:10.1007/s13659-012-0095-z)

Electronic Supplementary Material

Daphmacrodins A and B, alkaloids from *Daphniphyllum macropodum*

Ming-Ming CAO,[a,b] Hong-Ping HE,[a] Yu-Cheng GU,[c] Qiang ZHANG,[d] Xiao-Nian LI,[a] Guo-Ying ZUO,[e] Ying-Tong DI,[a] Chun-Mao YUAN,[a] Shun-Lin LI,[a] Yu ZHANG,[a,*] and Xiao-Jiang HAO[a,*]

[a]State Key Laboratory of Phytochemistry and Plant Resources in West China, Kunming Institute of Botany, Chinese Academy of Sciences, Kunming 650201, China

[b]University of Chinese Academy of Sciences, Beijing 100049, China

[c]Syngenta, Jealott's Hill International Research Centre, Bracknell, Berkshire, RG42 6EY, UK

[d]Northwest A&F University, Yangling 712100, China

[e]Research Center of Natural Medicine, Clinical School of Kunming General Hospital of Chengdu Military Command, Kunming 650032, China

Received 30 November 2012; Accepted 16 February 2013

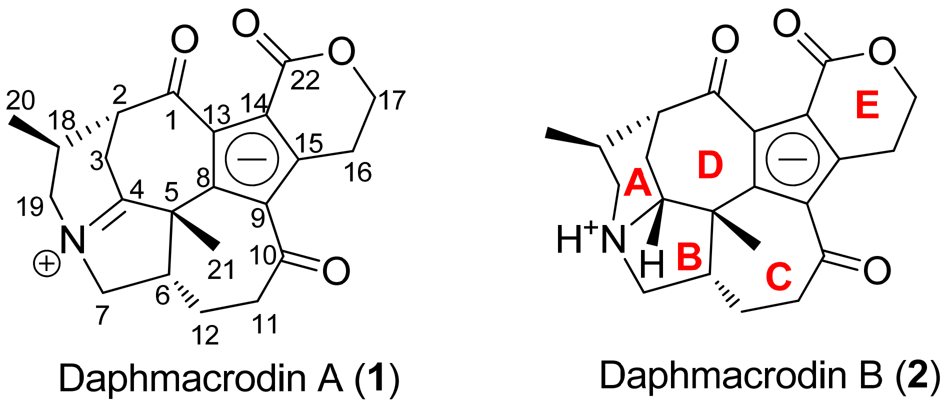

Structures of compounds **1** and **2**

*To whom correspondence should be addressed. E-mail:haoxj@mail.kib.ac.cn (X.J. Hao); zhangyu@mail.kib.ac.cn (Y. Zhang)

S1.1 ^{1}H NMR spectrum of daphmacrodin A (**1**) in DMSO-d_6

S1.2 ^{13}C NMR spectrum of daphmacrodin A **(1)** in DMSO-d_6

S1.3 HSQC spectrum of daphmacrodin A (**1**) in DMSO-d_6

S1.4 COSY spectrum of daphmacrodin A (**1**) in DMSO-d_6

S1.5 HMBC spectrum of daphmacrodin A (**1**) in DMSO-d_6

S1.6 ROESY spectrum of daphmacrodin A (**1**) in DMSO-d_6

S1.7 ESIMS and HRESIMS spectrums of daphmacrodin A (**1**)

S1.8 IR spectrum of daphmacrodin A (**1**)

S1.9 ECD spectrum of daphmacrodin A (**1**) in methanol

S1.10 X-ray crystal structure of daphmacrodin A **(1)**

S2.1 ^{1}H NMR spectrum of daphmacrodin B (**2**) in DMSO-d_6

S2.2 ^{13}C NMR spectrum of daphmacrodin B **(2)** in DMSO-d_6

S2.3 HSQC spectrum of daphmacrodin B (**2**) in DMSO-d_6

S2.4 COSY spectrum of daphmacrodin B (**2**) in DMSO-d_6

S2.5 HMBC spectrum of daphmacrodin B (**2**) in DMSO-d_6

S2.6 ROESY spectrum of daphmacrodin B (**2**) in DMSO-d_6

S2.7 ESIMS and HRESIMS spectrums of daphmacrodin B (**2**)

S2.8 IR spectrum of daphmacrodin B (**2**)

S2.9 ECD spectrum of daphmacrodin B (**2**) in methanol

S1.1 ^{1}H NMR spectrum of daphmacrodin A (**1**) in DMSO-d_6

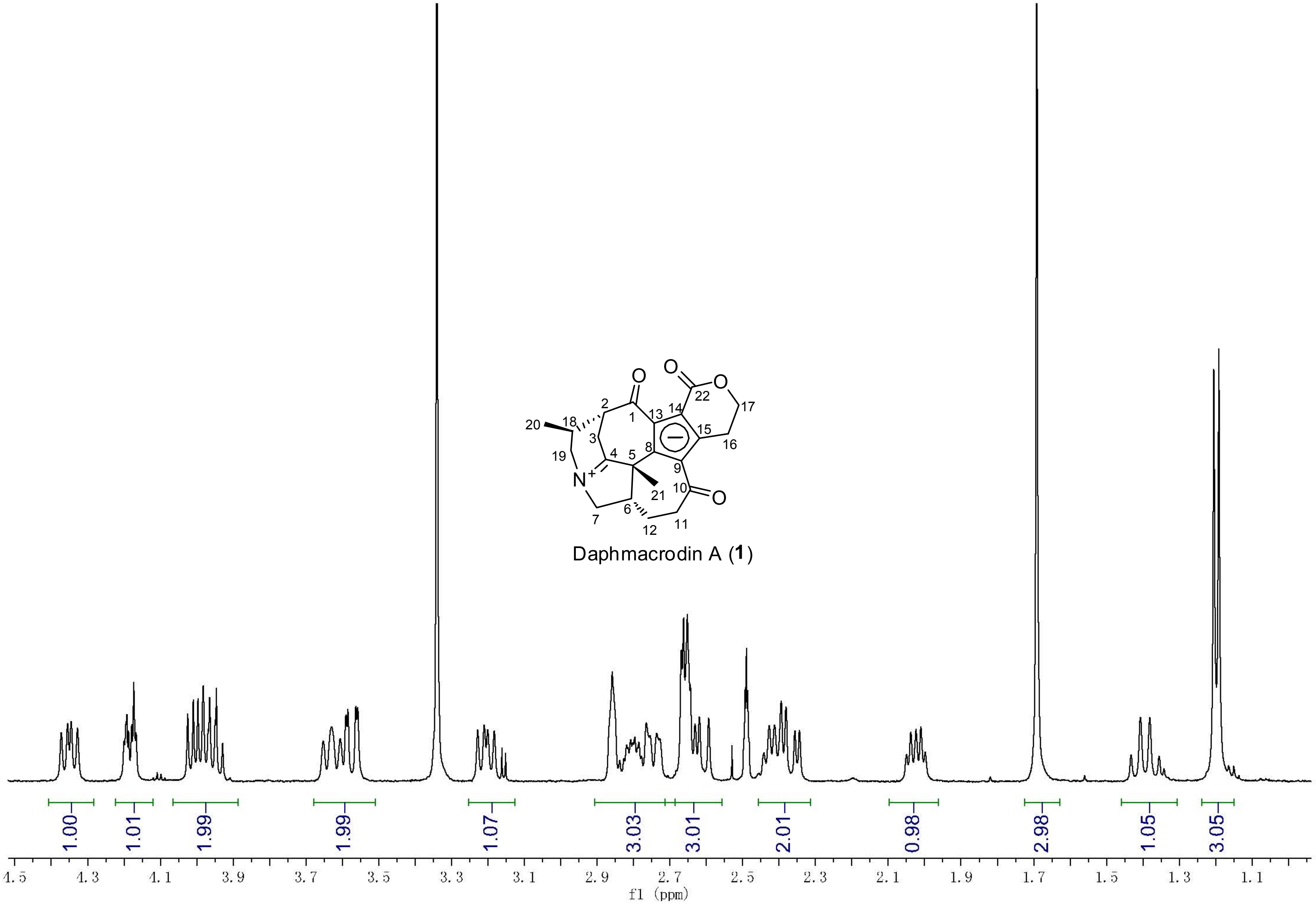

S1.2 ^{13}C NMR spectrum of daphmacrodin A **(1)** in DMSO-d_6

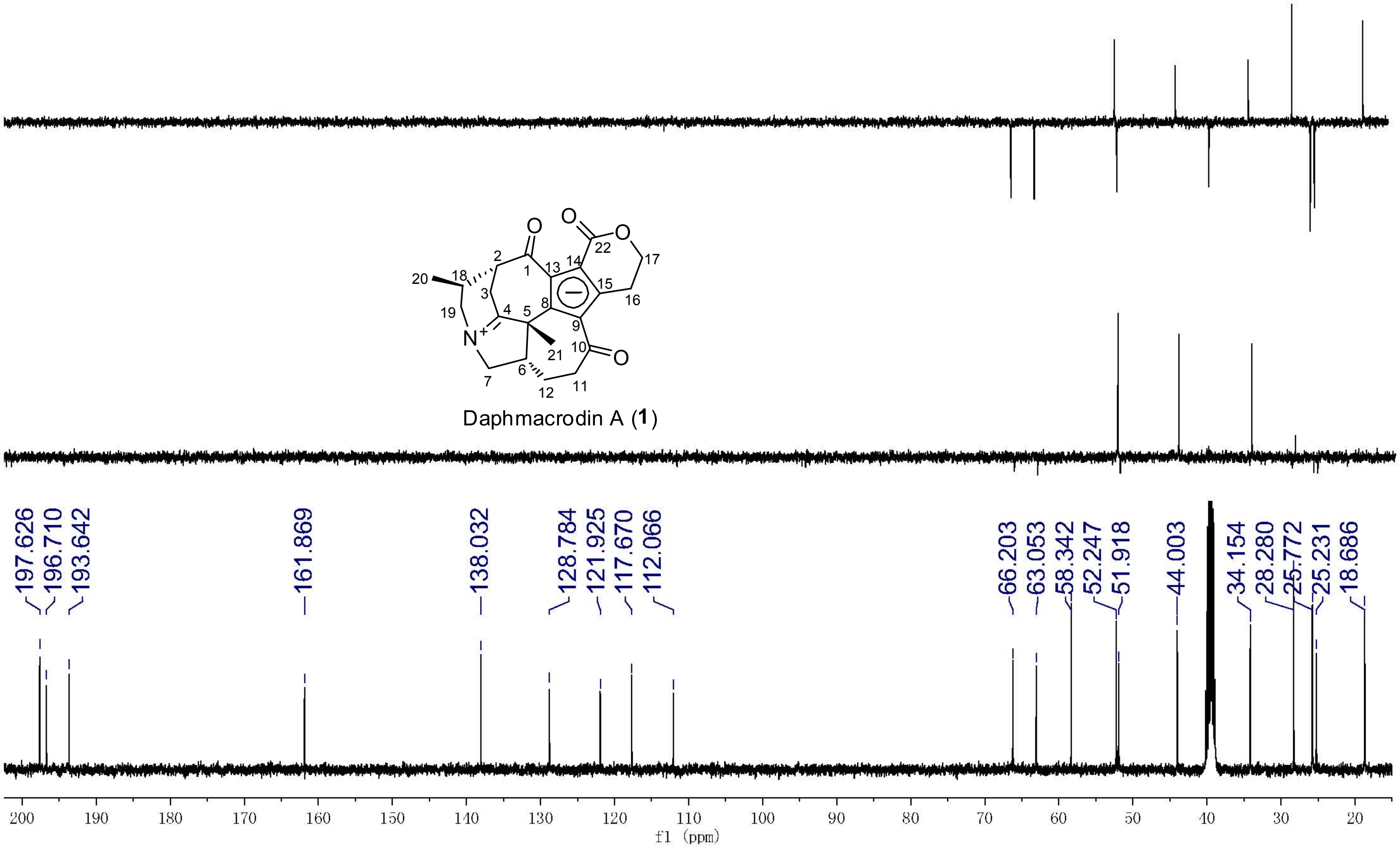

S1.3 HSQC spectrum of daphmacrodin A (**1**) in DMSO-d_6

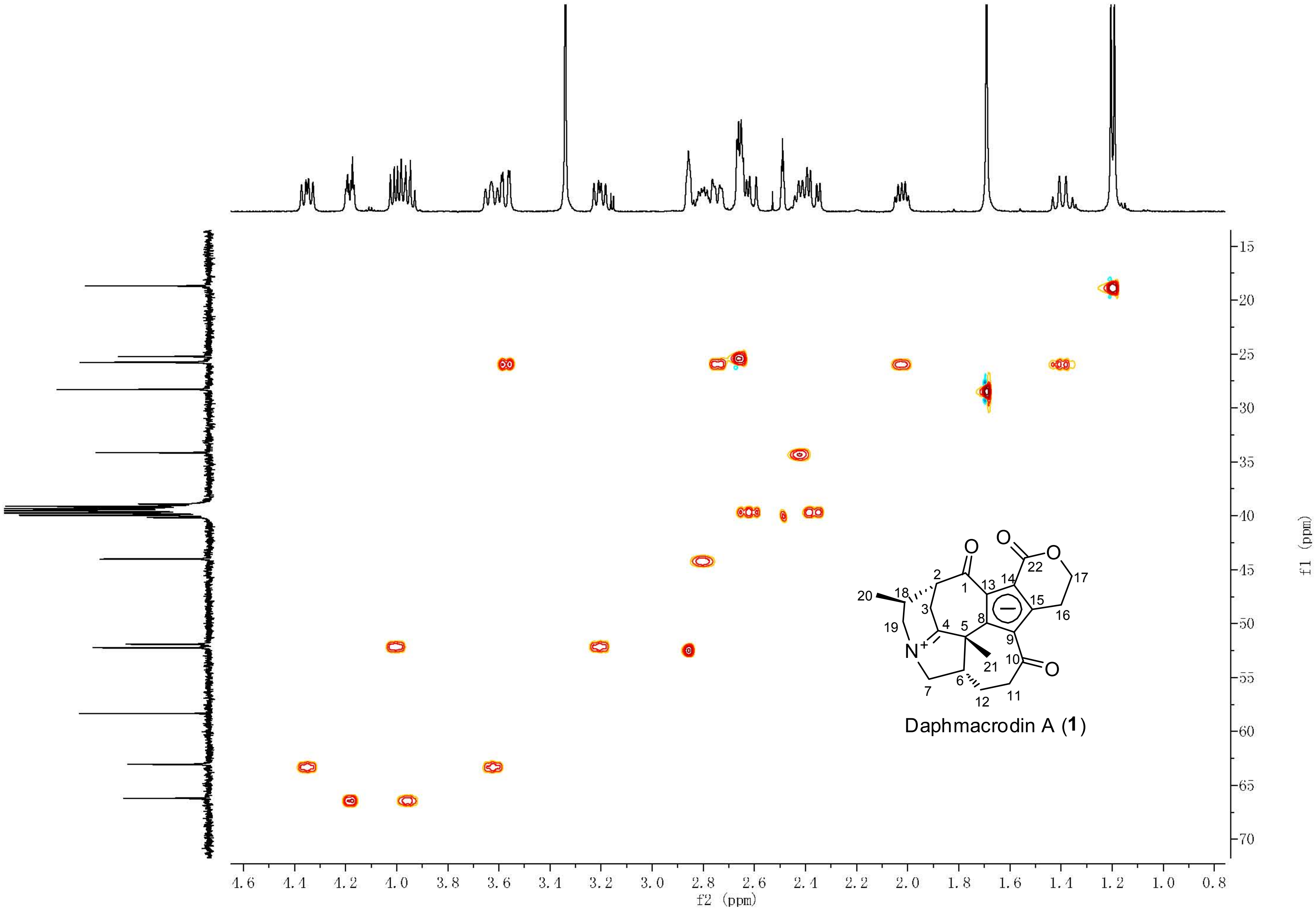

S1.4 COSY spectrum of daphmacrodin A (**1**) in DMSO-d_6

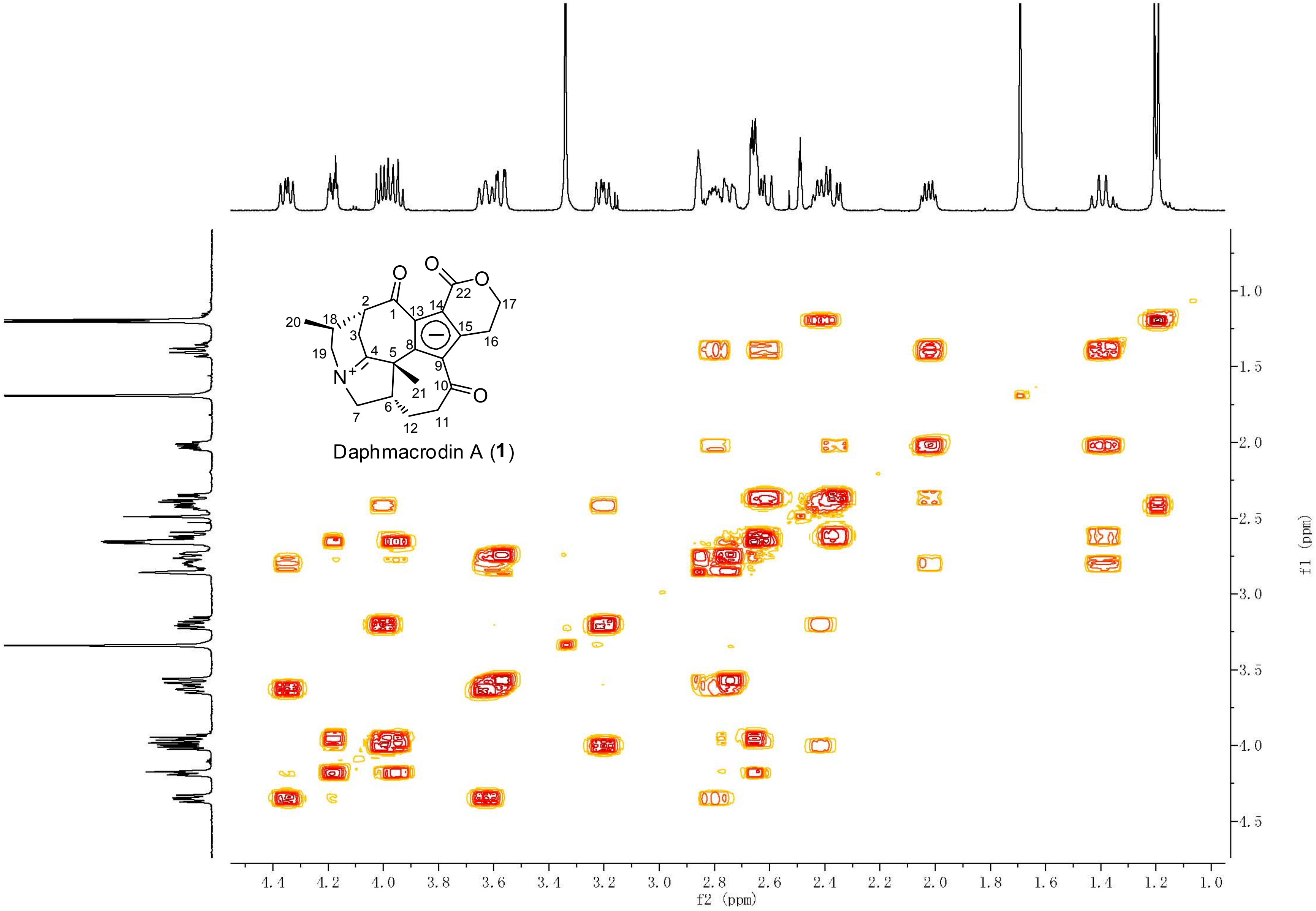

S1.5 HMBC spectrum of daphmacrodin A (**1**) in DMSO-d_6

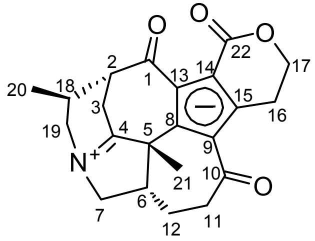

Daphmacrodin A (**1**)

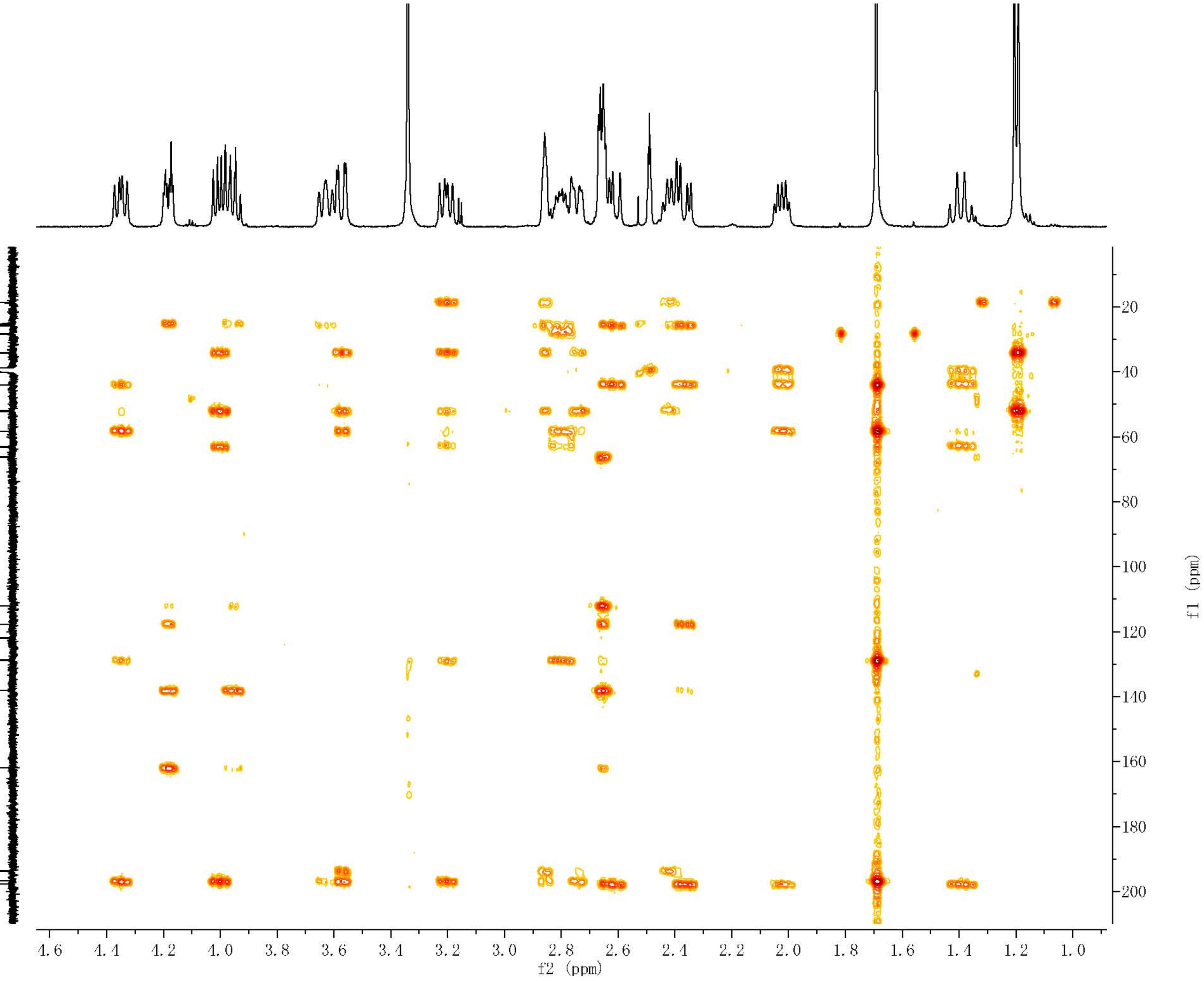

S1.6 ROESY spectrum of daphmacrodin A (**1**) in DMSO-d_6

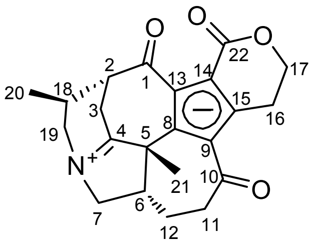

Daphmacrodin A (**1**)

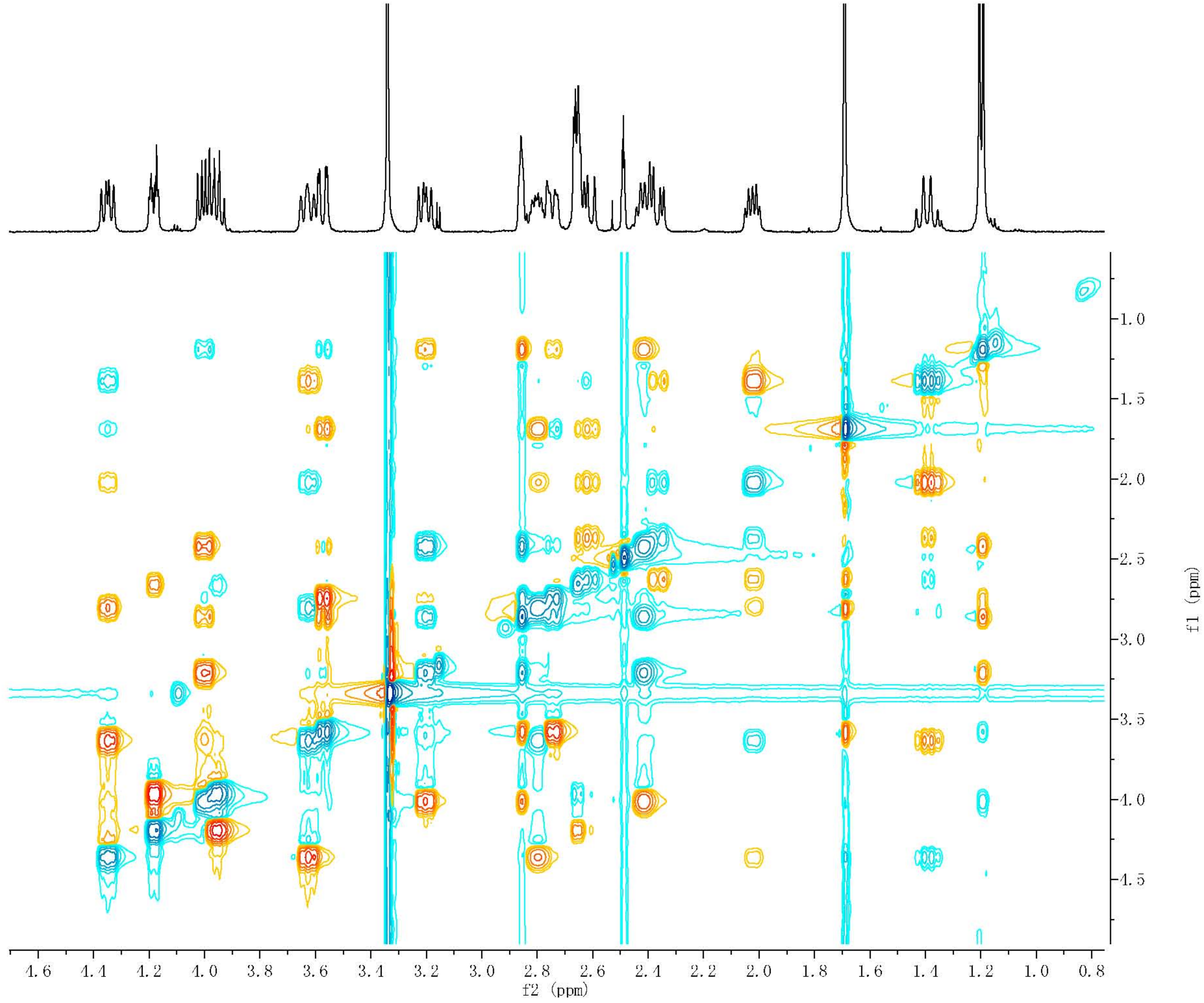

S1.7 ESIMS and HRESIMS spectrums of daphmacrodin A (**1**)

Mass Spectrum List Report

Analysis Info

Analysis Name	D:\DATA\2011file\1112\111201\hcm-46a00.d	Acquisition Date	11/25/2011 1:40:21 PM
Method	DEF_MS .M	Operator	quyan
Sample Name	hcm-46a	Instrument	HCT

Acquisition Parameter

Ion Source Type	ESI	Ion Polarity	Positive	Alternating Ion Polarity	off
Mass Range Mode	Ultra Scan	Scan Begin	100 m/z	Scan End	900 m/z
Capillary Exit	200.0 Volt	Skimmer	40.0 Volt	Trap Drive	32.9
Accumulation Time	38098 µs	Averages	5 Spectra	Auto MS/MS	off

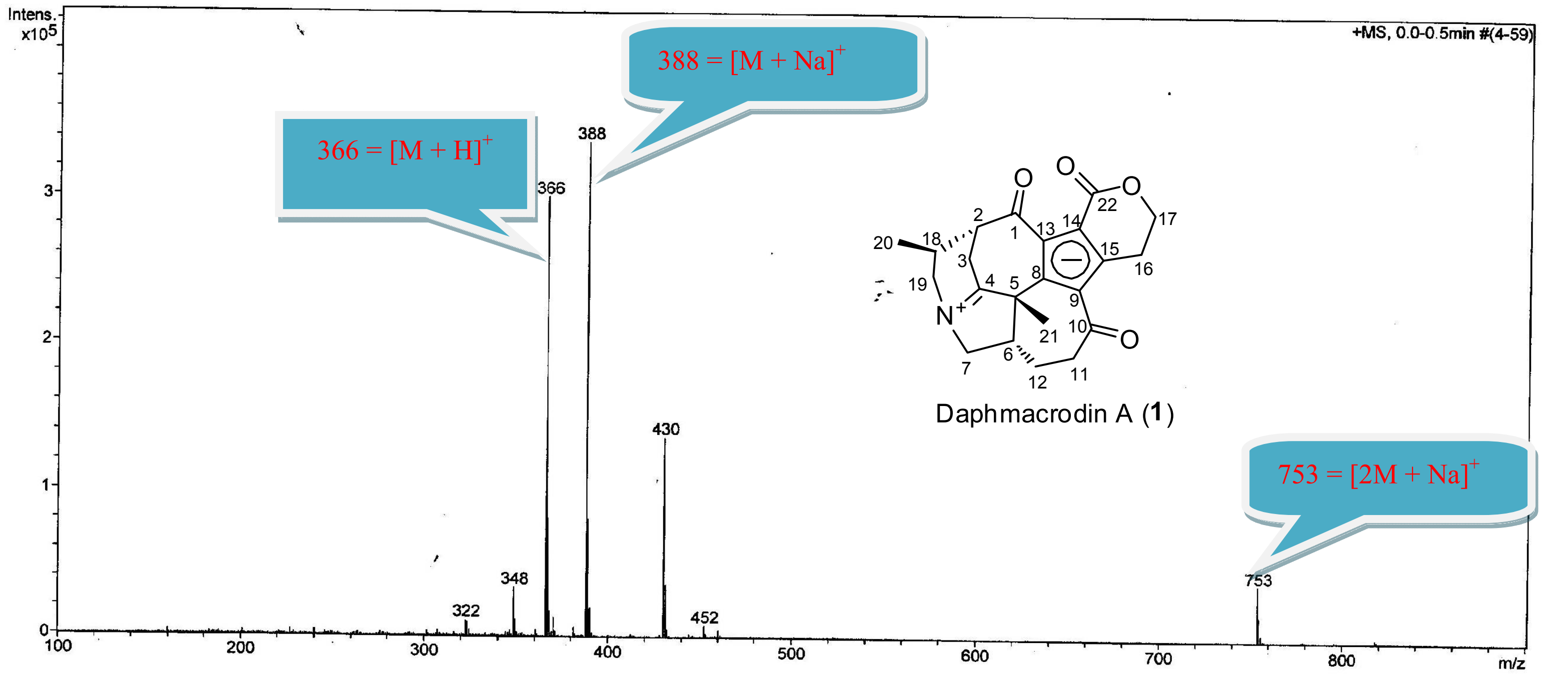

Acq. Date: Thursday, February 16, 2012 Acq. Time: 15:21

Sample Name: 120217ESIA hcm-46a

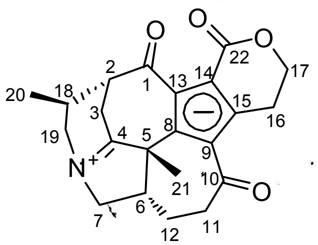

Daphmacrodin A (**1**)

Elemental composition calculator

Target m/z: +388.1528 amu
Tolerance: +10.0000 ppm
Result type: Elemental
Max num of results: 1000
Min DBE: -10.0000 Max DBE: +60.0000
Electron state: OddAndEven
Num of charges: 0
Add water: N/A
Add proton: N/A
File Name: 120217ESIA hcm-46a.wiff

Acq. Date: Thursday, February 16, 2012 Acq. Time: 15:21

Sample Name: 120217ESIA hcm-46a

	Elements	Min Number	Max Number
11	O	1	4
12	P	0	0
13	Pt	0	0
14	S	0	0
15	Si	0	0

	Formula	Calculated m/z (amu)	mDa Error	PPM Error	DBE
1	C22 H23 N O4 Na	388.1524	0.3218	0.8290	11.5

S1.8 IR spectrum of daphmacrodin A (**1**)

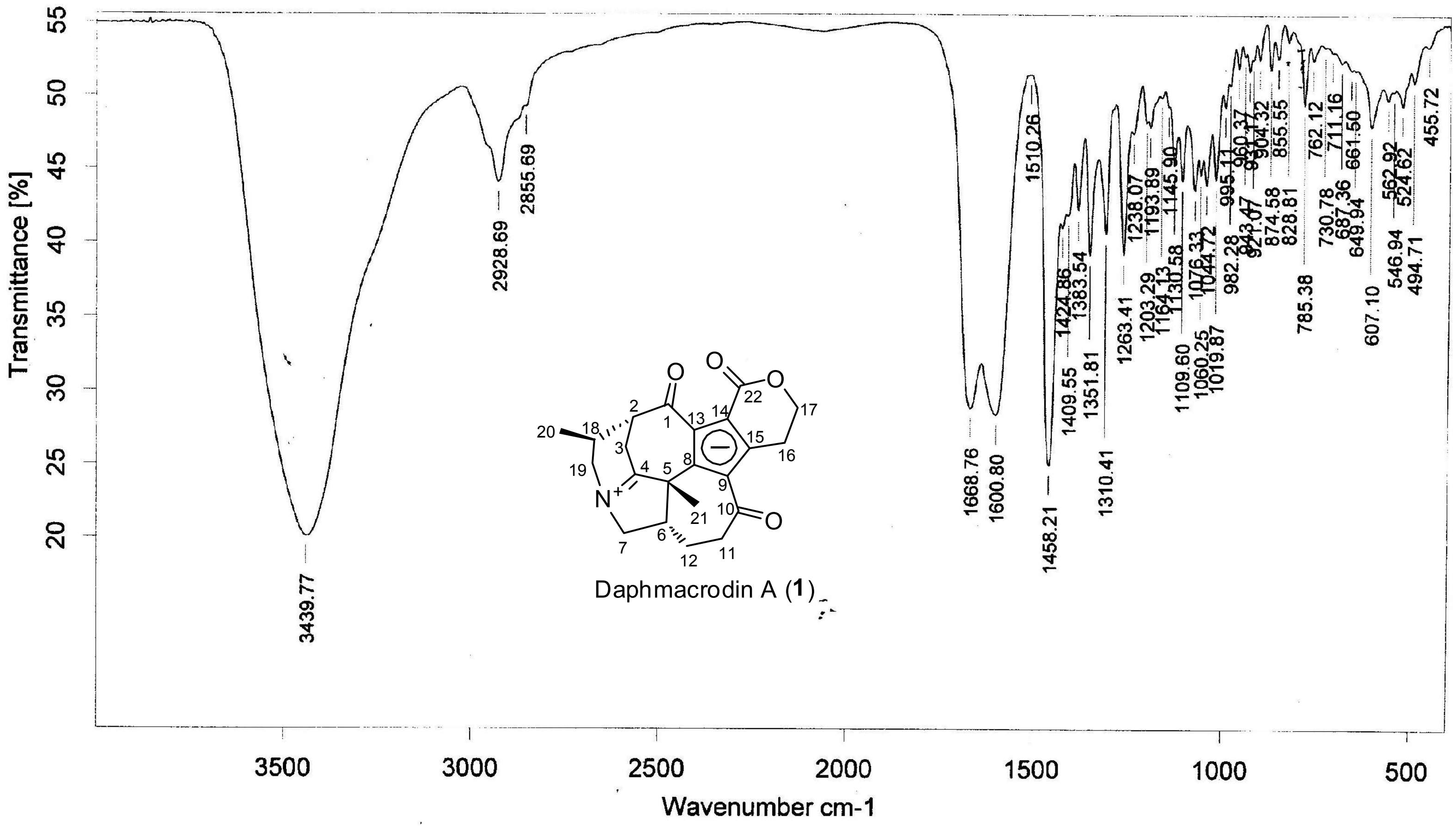

Sample : hcm-46a		Frequency Range : 399.246 - 3996.32	Measured on : 16/02/2012
Technique : KBr压片	Resolution : 4	Instrument : Tensor27	Sample Scans : 16
Customer : 120217IR0	Zerofilling : 2	Acquisition : Double Sided,For	

S1.9 ECD spectrum of daphmacrodin A (**1**) in methanol

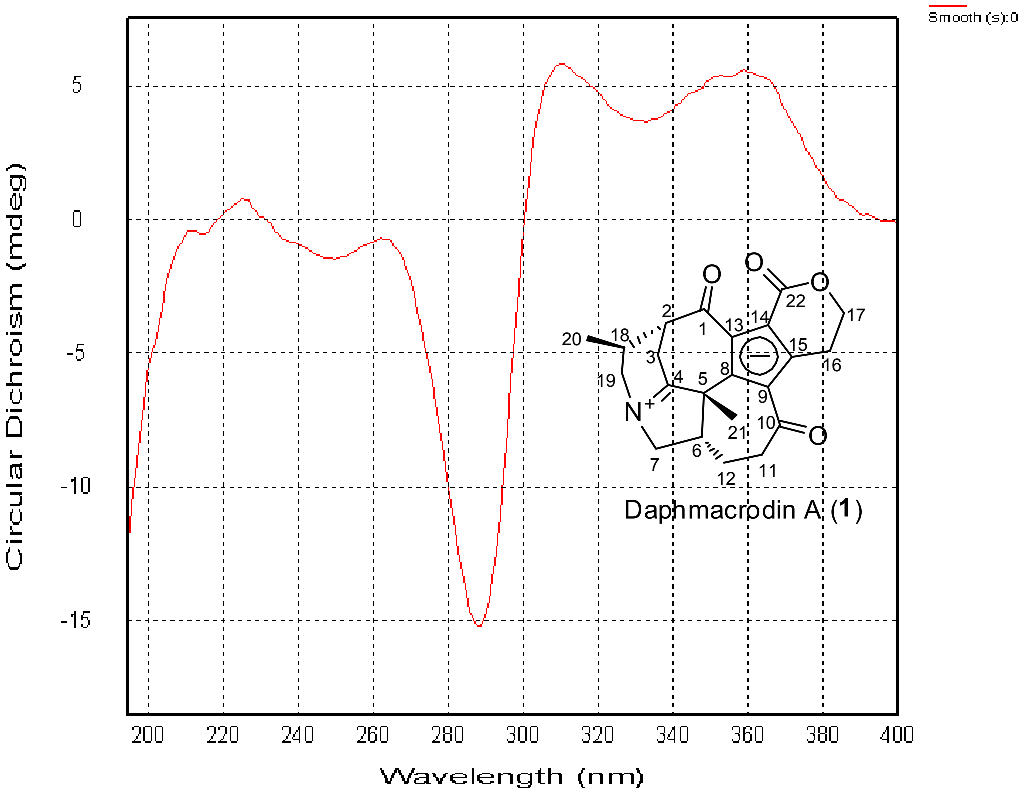

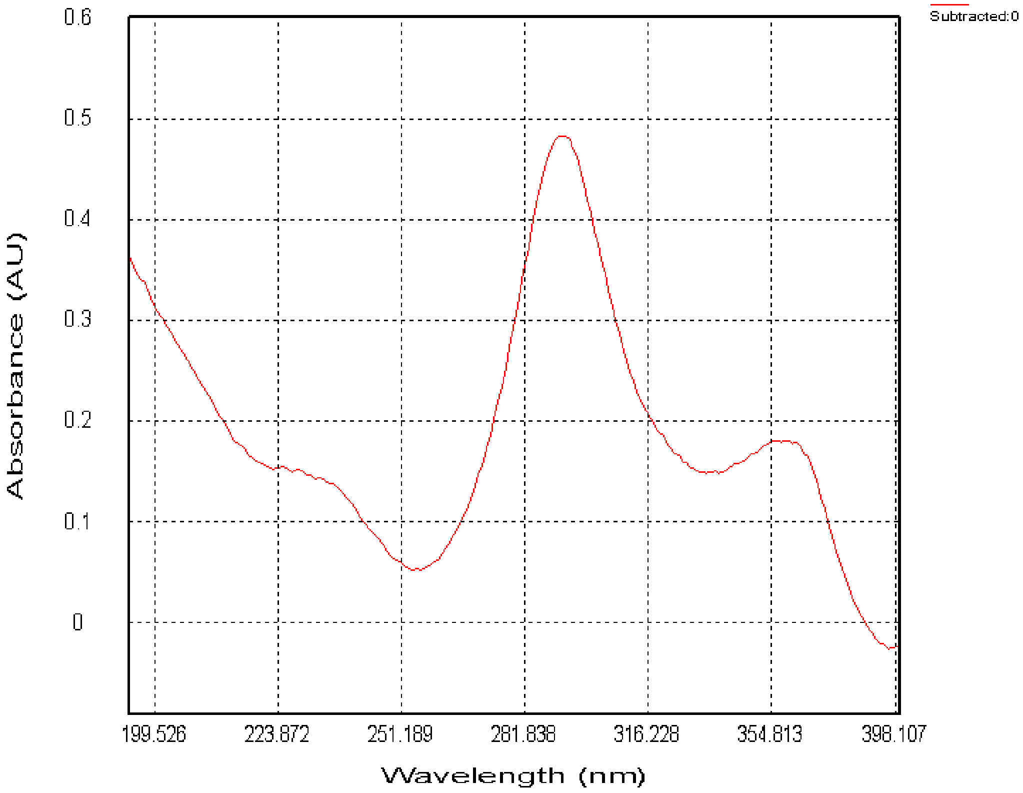

S1.10 X-ray crystal structure of daphmacrodin A **(1)**

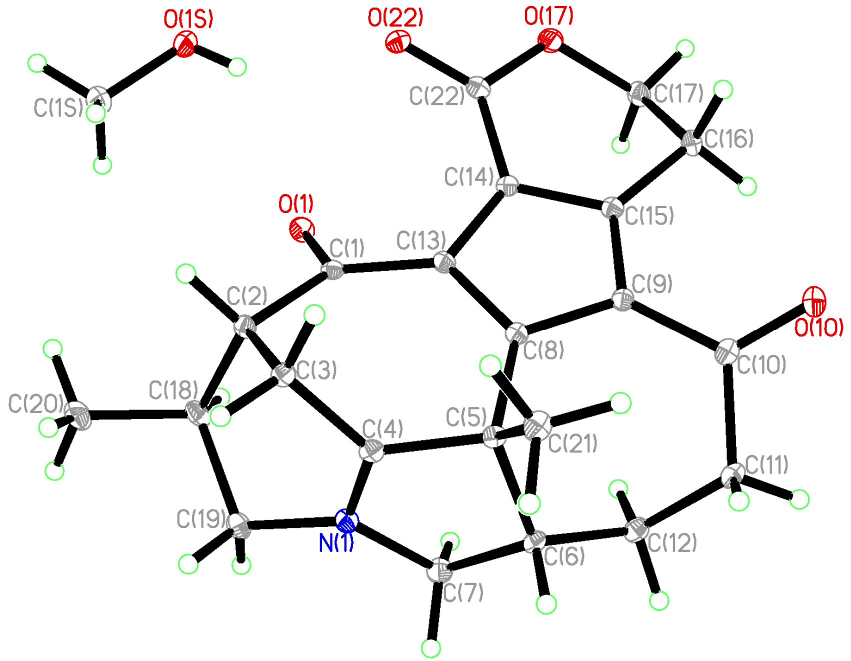

Crystal data and structure refinement for daphmacrodin A **(1)**	
Empirical formula	$C_{23}H_{27}NO_5$
Formula weight	397.46
Temperature	100(2) K
Wavelength	0.71073 Å
Crystal system, space group	Monoclinic, P 21
F(000)	424
Reflections collected / unique	10303 / 5321 [R(int) = 0.0232]
Refinement method	Full-matrix least-squares on F^2
R indices (all data)	R1 = 0.0485, wR2 = 0.1404
Absolute structure parameter	-0.5(10)

S2.1 ^{1}H NMR spectrum of daphmacrodin B (**2**) in DMSO-d_6

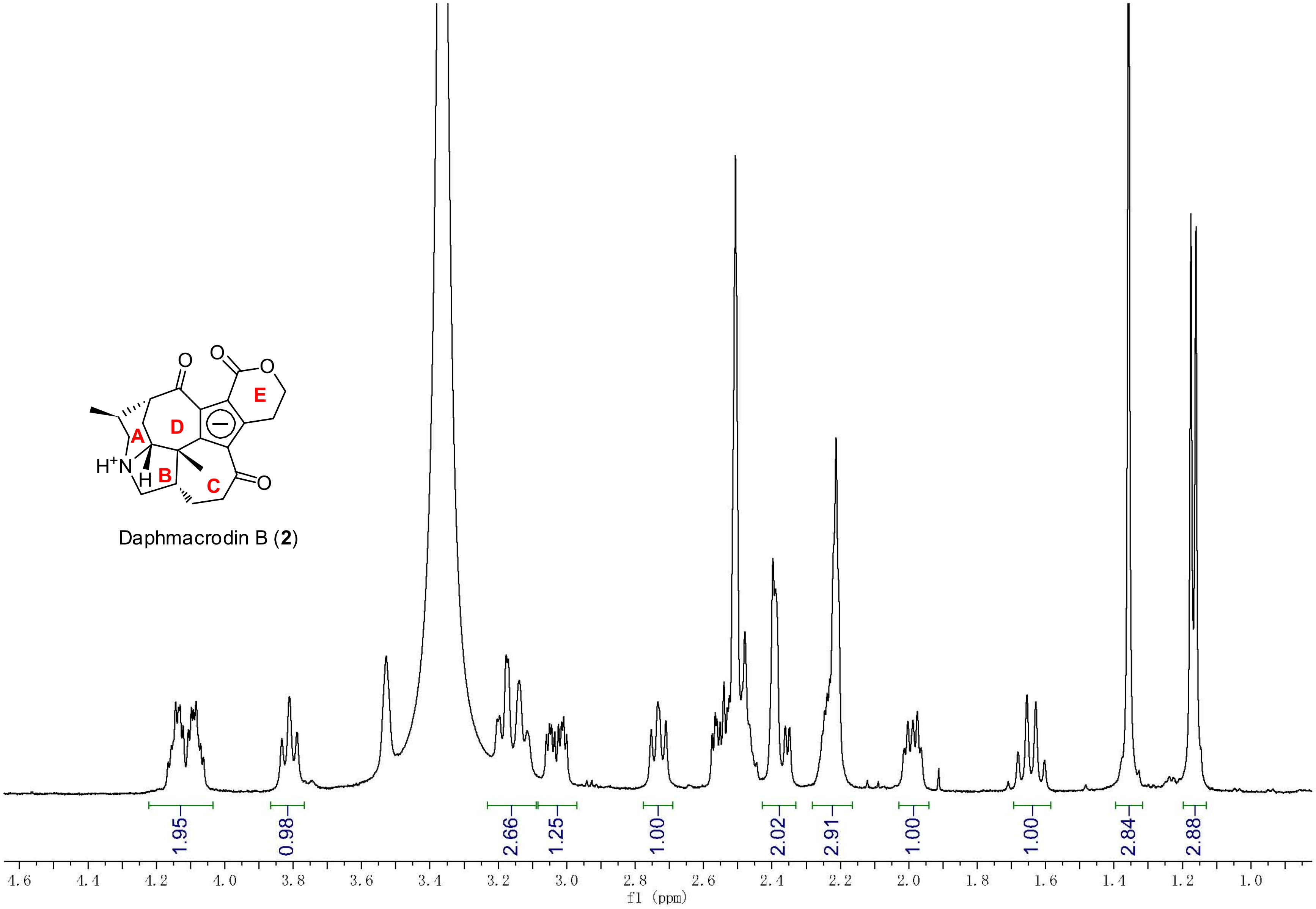

S2.2 ^{13}C NMR spectrum of daphmacrodin B **(2)** in DMSO-d_6

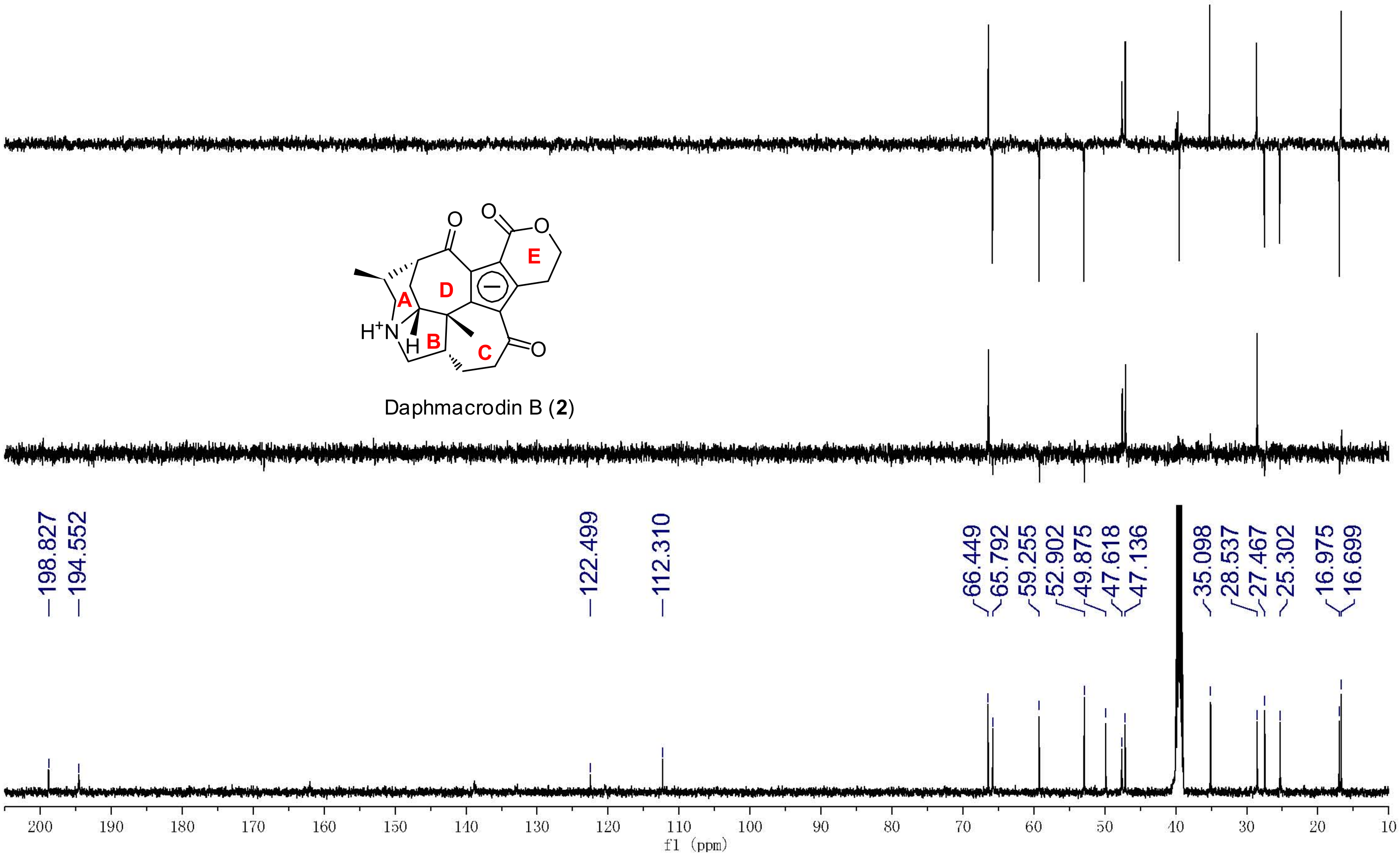

S2.3 HSQC spectrum of daphmacrodin B (**2**) in DMSO-d_6

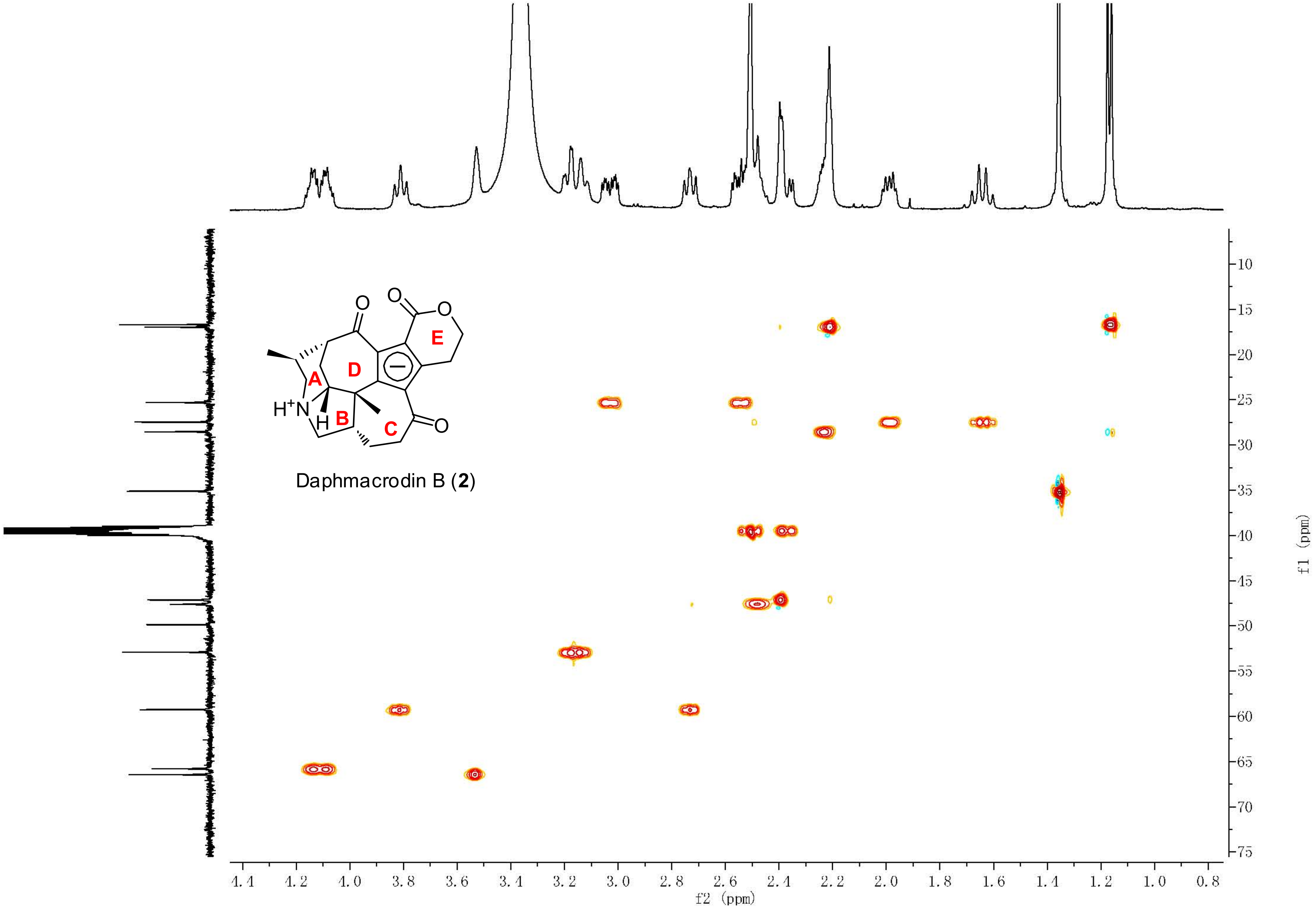

S2.4 COSY spectrum of daphmacrodin B (**2**) in DMSO-d_6

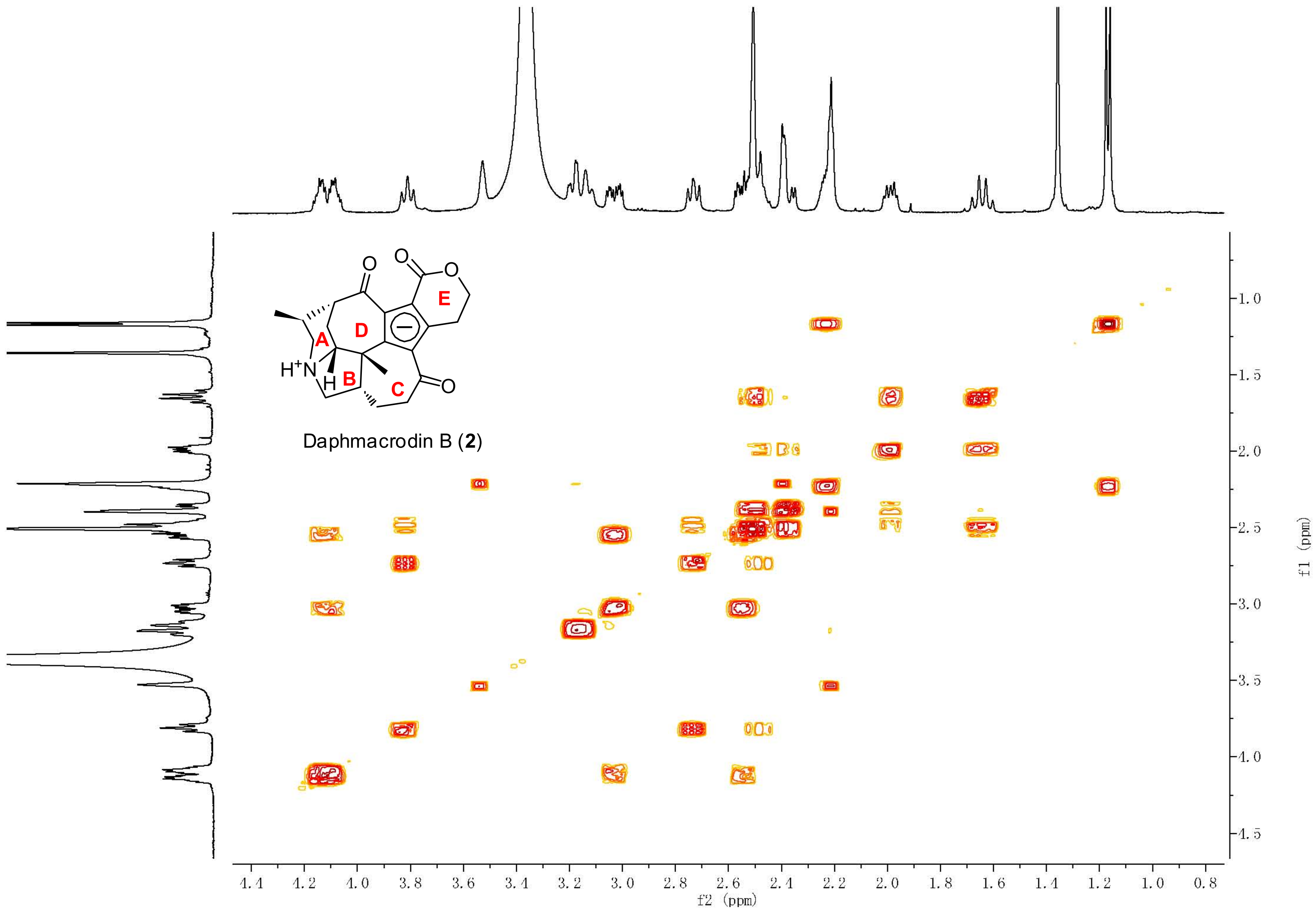

S2.5 HMBC spectrum of daphmacrodin B (**2**) in DMSO-d_6

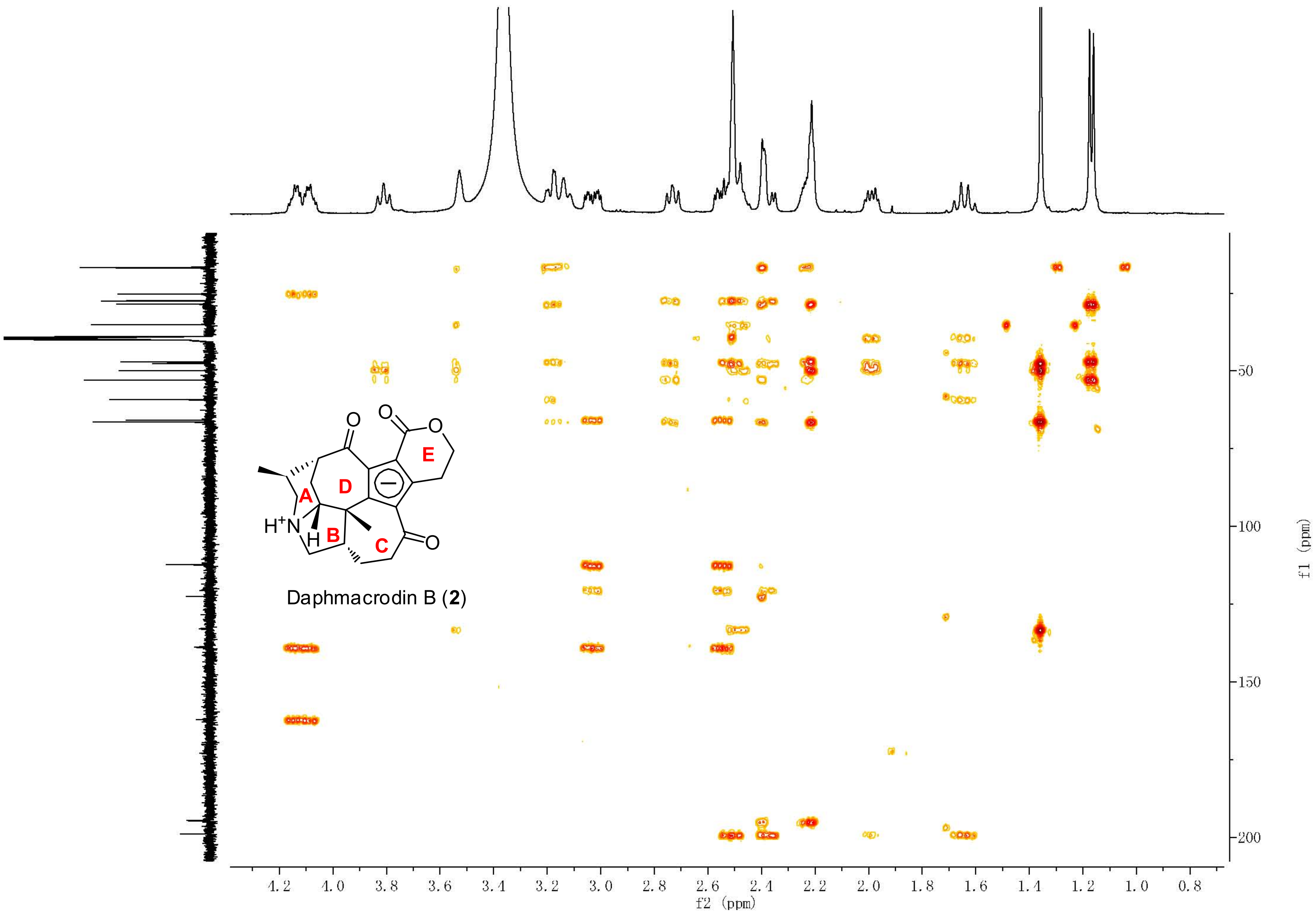

S2.6 ROESY spectrum of daphmacrodin B (**2**) in DMSO-d_6

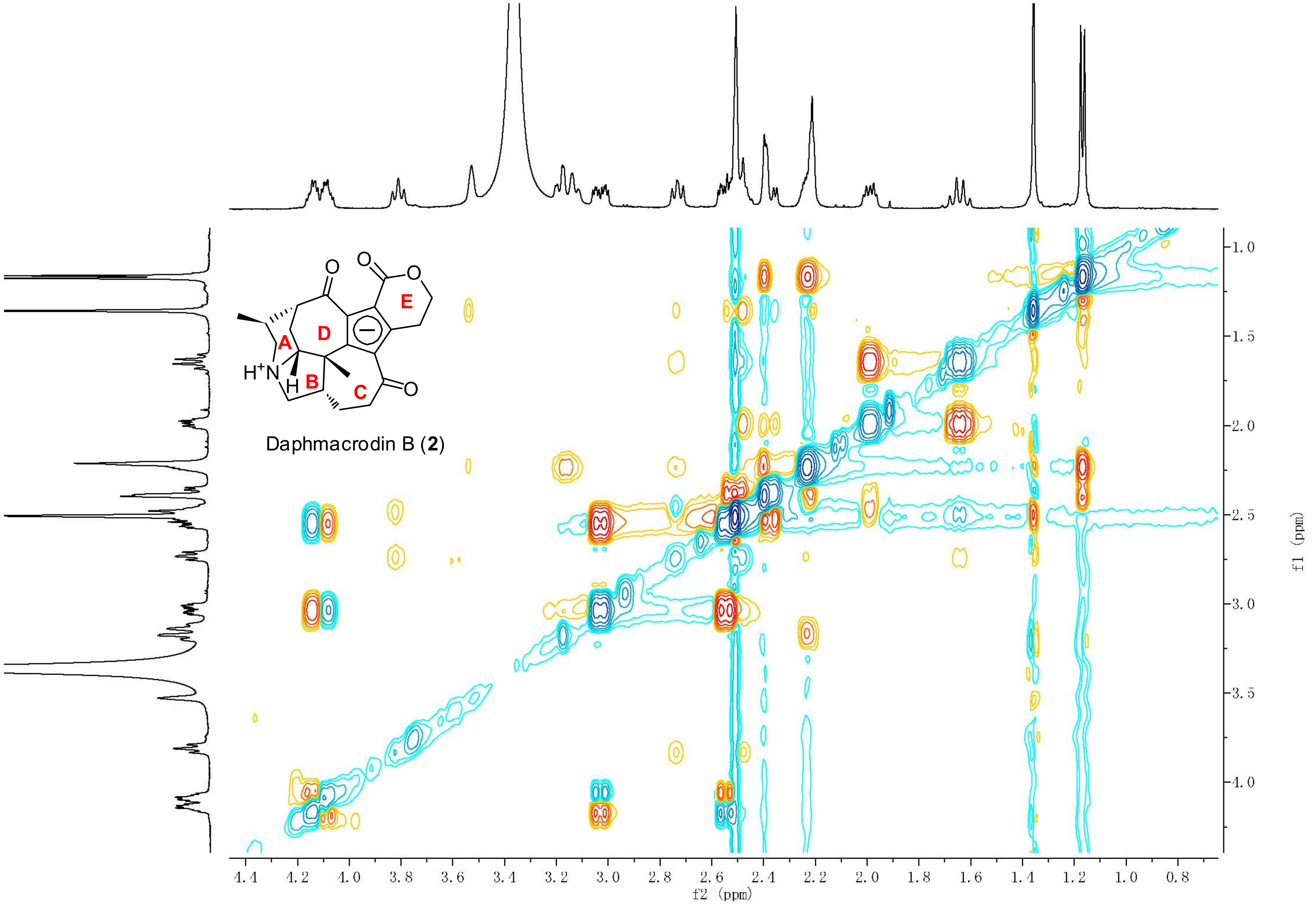

S2.7 ESIMS and HRESIMS spectrums of daphmacrodin B (**2**)

Mass Spectrum List Report

Analysis Info

Analysis Name	D:\DATA\2011file\1112\111201\hcm-4301.d	Acquisition Date	11/25/2011 1:17:23 PM
Method	DEF_MS .M	Operator	quyan
Sample Name	hcm-43	Instrument	HCT

Acquisition Parameter

Ion Source Type	ESI	Ion Polarity	Positive	Alternating Ion Polarity	off
Mass Range Mode	Ultra Scan	Scan Begin	100 m/z	Scan End	800 m/z
Capillary Exit	113.5 Volt	Skimmer	40.0 Volt	Trap Drive	32.9
Accumulation Time	100000 µs	Averages	5 Spectra	Auto MS/MS	off

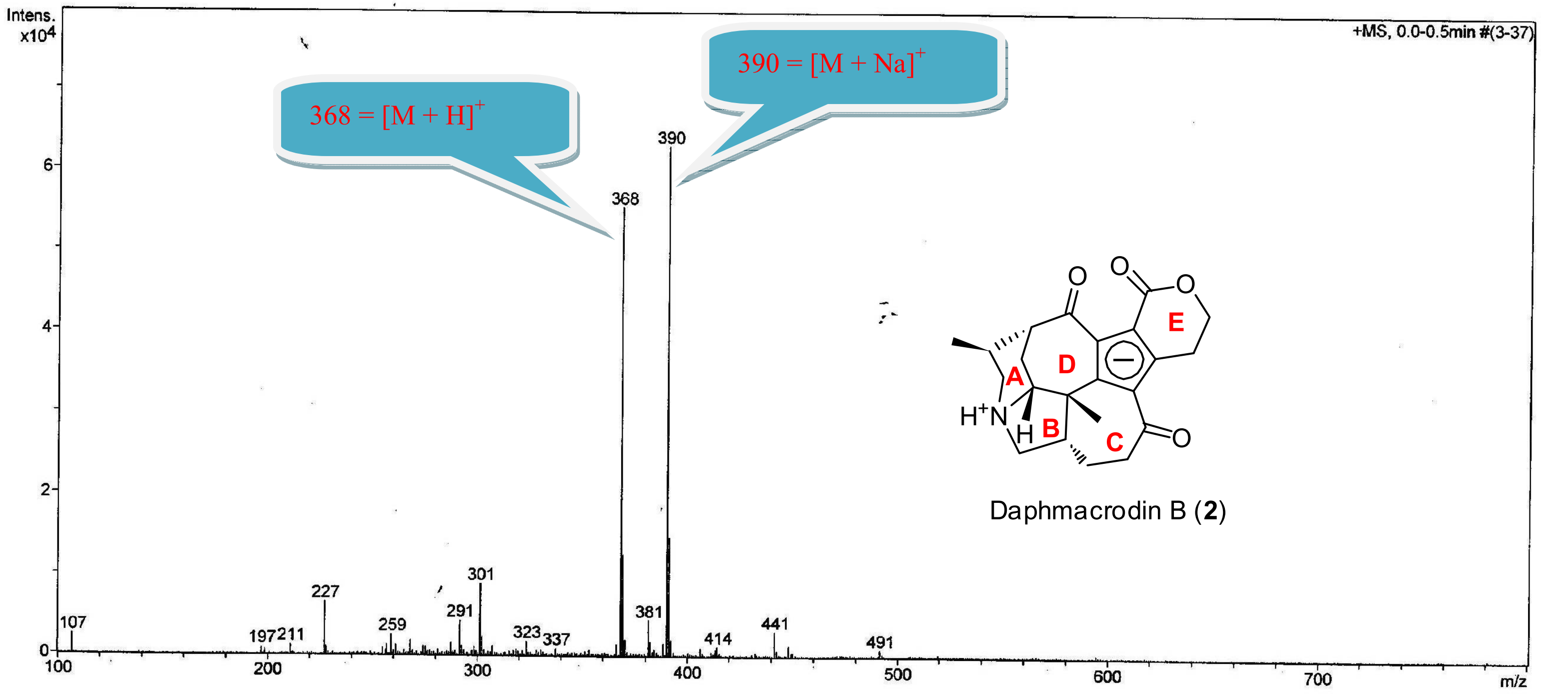

Daphmacrodin B (**2**)

Acq. Date: Thursday, February 16, 2012 Acq. Time: 15:26

Sample Name: 120217ESIA hcm-43

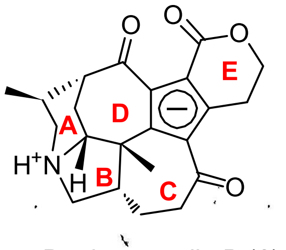

Daphmacrodin B (**2**)

Elemental composition calculator

Target m/z:	+368.1861	amu	
Tolerance:	+10.0000	ppm	
Result type:	Elemental		
Max num of results:	1000		
Min DBE:	-10.0000	Max DBE:	+60.0000
Electron state:	OddAndEven		
Num of charges:	0		
Add water:	N/A		
Add proton:	N/A		
File Name:	120217ESIA	hcm-43.wiff	

Acq. Date: Thursday, February 16, 2012 Acq. Time: 15:26

Sample Name: 120217ESIA hcm-43

	Elements	Min Number	Max Number
11	O	1	4
12	P	0	0
13	Pt	0	0
14	S	0	0
15	Si	0	0

	Formula	Calculated m/z (amu)	mDa Error	PPM Error	DBE
1	C22 H26 N O4	368.1861	-0.0836	-0.2270	10.5

S2.8 IR spectrum of daphmacrodin B (**2**)

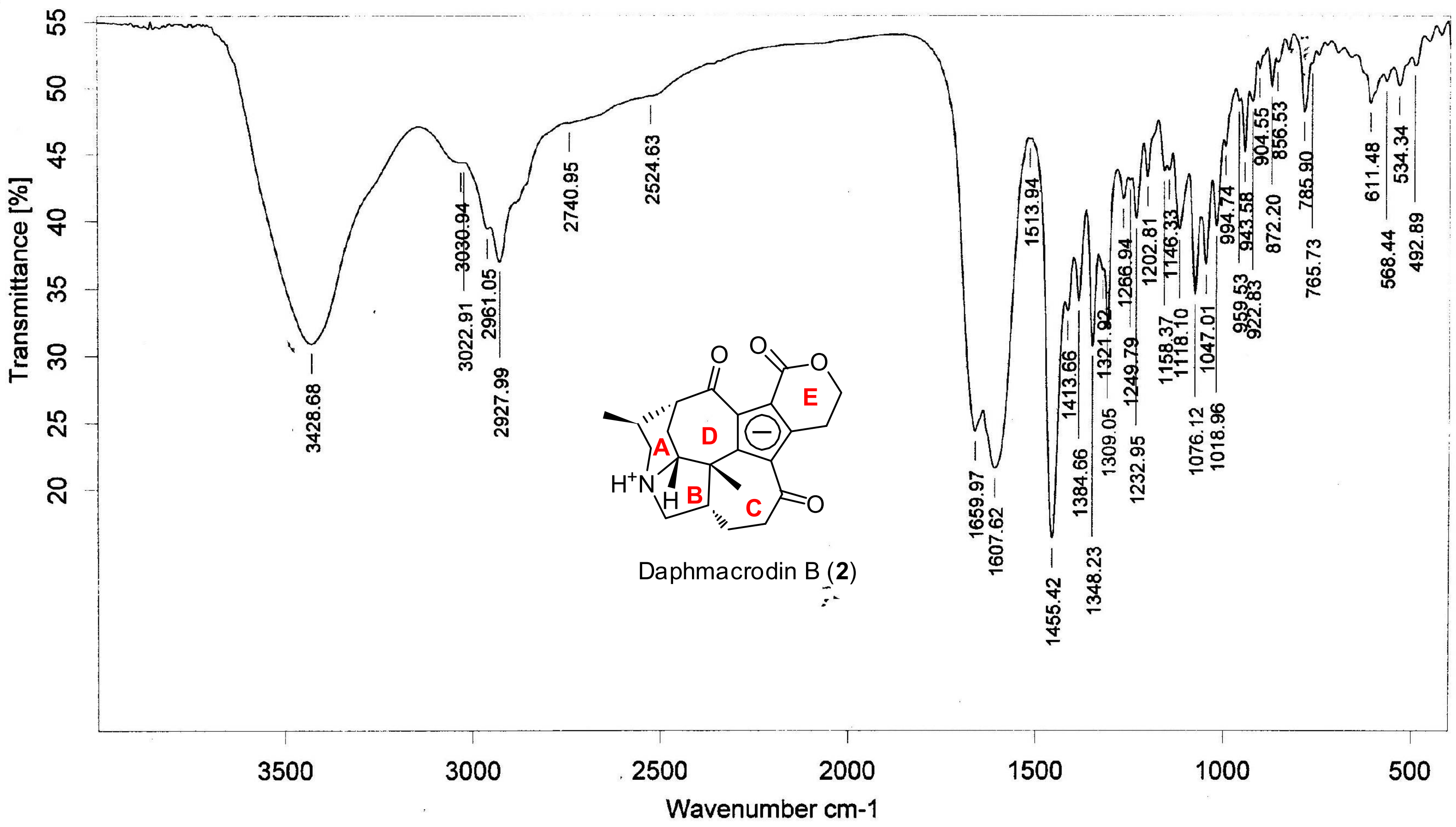

Sample : hcm-43	Frequency Range : 399.246 - 3996.32	Measured on : 16/02/2012	
Technique : KBr压片	Resolution : 4	Instrument : Tensor27	Sample Scans : 16
Customer : 120217IR1	Zerofilling : 2	Acquisition : Double Sided,For	

S2.9 ECD spectrum of daphmacrodin B (**2**) in methanol

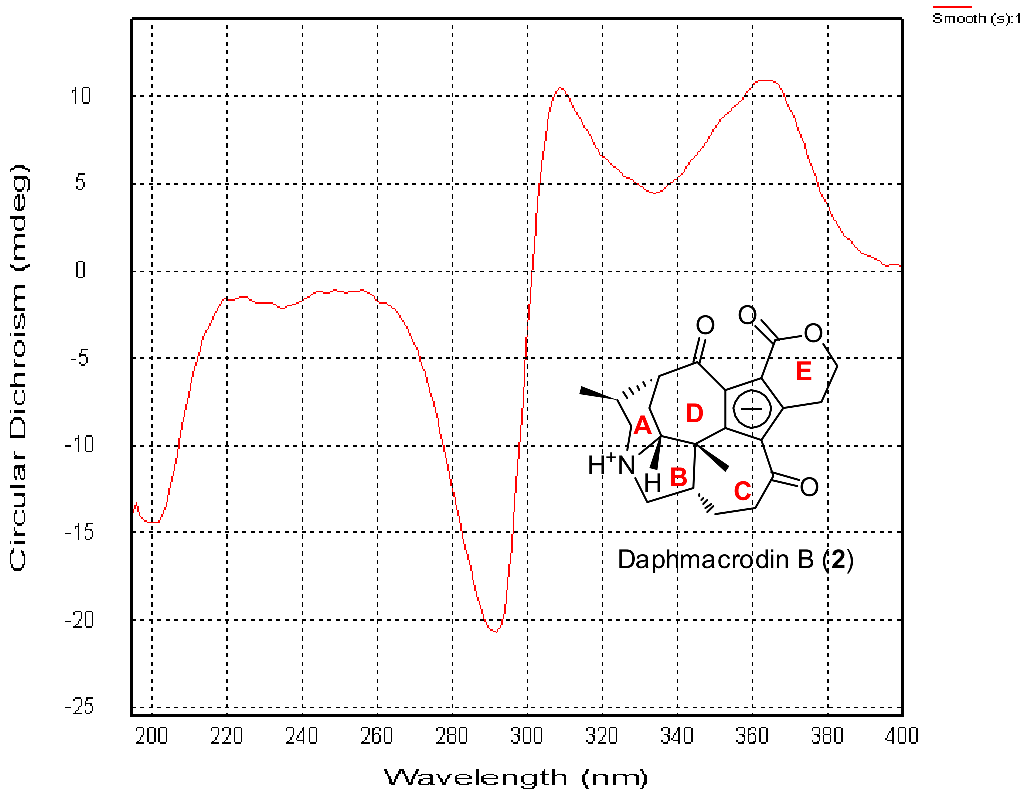

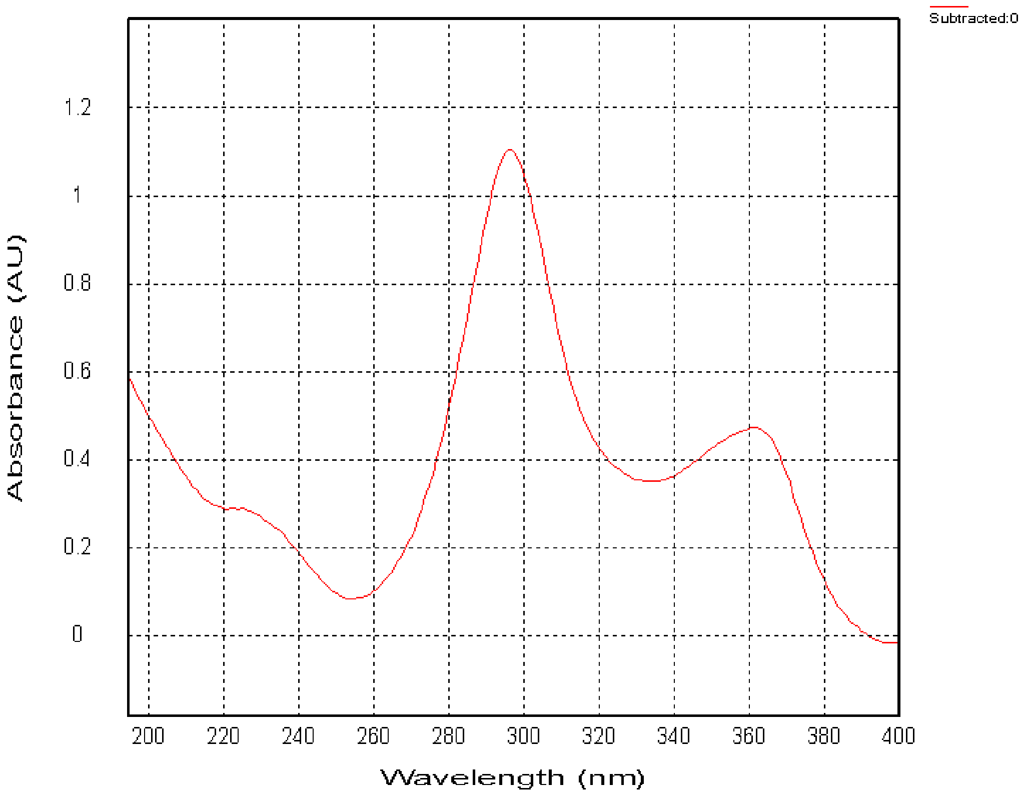

Supplement: Supplementary file 1 — Supplementary material, approximately 4.91 MB. [file 13659_2012_95_MOESM1_ESM.pdf]
